# The impact of generative AI’S information delivery methods on emotional exhaustion among bullying roles in the medical workplace

**DOI:** 10.3389/fpubh.2025.1649342

**Published:** 2025-09-15

**Authors:** Lihong Deng, Dajun Yang, Gongzhuoran Liang, Chang Hu, Pengcheng Zhang

**Affiliations:** ^1^College of Management, North Sichuan Medical College, Nanchong, China; ^2^Key Laboratory of Digital Intelligent Disease Surveillance and Health Governance, North Sichuan Medical College, Nanchong, China; ^3^Sichuan Provincial Primary Health Service Development Research Center, North Sichuan Medical College, Nanchong, China; ^4^College of Physical Education, Jiangxi Normal University, Nanchang, China

**Keywords:** generative AI, information delivery methods, workplace bullying, bullying roles, emotional exhaustion, bystander, victim

## Abstract

Workplace bullying is closely related to poor work states. Previous studies have primarily explored the binary relationship between perpetrators and victims, with limited research examining the emotional exhaustion of bullying roles from the perspectives of victims and bystanders. Therefore, this study recruited 597 participants and conducted a scenario-based experiment to investigate whether generative AI can alleviate the poor work states of bullying roles in the medical workplace, thereby demonstrating the interaction between generative AI’s information delivery methods and bullying roles in relation to emotional exhaustion. The results showed that bullying roles in the medical workplace significantly influence emotional exhaustion, with victims experiencing significantly higher levels than bystanders. Moreover, generative AI’s information delivery methods can effectively moderate the work states of victims. Thus, this study advances the field of human-computer interaction by shifting its focus from functional adaptation to emotional ecology. It also provides empirical evidence from medical scenarios for the uncanny valley theory. Furthermore, this research lays a theoretical foundation for the design of emotional interaction functions in medical AI systems.

## 1 Introduction

Workplace bullying refers to a pattern of behavior where individuals are subjected to intentional humiliation, exclusion, or attacks in the workplace over a prolonged period (1). In recent years, as societal emphasis on mental health has increased, workplace bullying has become a focal point of interdisciplinary research (2, 3). On one hand, public awareness of workplace bullying has significantly improved due to its destructive effects on reducing employee well-being and increasing turnover rates (4, 5). For instance, Cullinan et al. (6) found that workplace bullying can increase employees’ risk of depression and lead to increased annual productivity losses. On the other hand, the covert and high-frequency nature of workplace bullying has made it more challenging to govern (7), particularly in high-pressure industries such as healthcare. Notably, workplace bullying in the medical field is particularly severe. According to the International Labour Organization, a significant number of healthcare professionals have experienced prolonged bullying, far exceeding other occupational groups (8). This high incidence is closely related to the high-intensity and high-risk nature of the medical field (9). Specifically, the shift system, life-or-death pressures, and resource competition in the medical field have intensified interpersonal conflicts (10–12). However, existing research has primarily focused on the impact of bullying behavior on organizational performance (13), while neglecting an in-depth exploration of the psychological depletion mechanisms of victims. For example, Rodwell and Demir (14) confirmed that bullying weakens career commitment through emotional exhaustion as a mediator but did not distinguish between the differential reaction pathways of victims and bystanders. Meanwhile, previous studies have mostly analyzed the factors influencing workplace bullying (15) but have not further conducted field experiments to investigate the effectiveness of interventions for workplace bullying. Therefore, this study aims to explore effective interventions for workplace bullying in the medical field through field experiments.

Previous research has emphasized that the roles in workplace bullying primarily include victims, perpetrators, and bystanders, with the complexity lying in the dynamic differentiation of roles and context specificity (16–18). Based on role interaction theory, bystanders may transform from moral disengagement into implicit accomplices or become protectors under organizational intervention (19, 20), while victims’ psychological resilience differences may lead them from passive endurance to active coping (21, 22). Both victims and bystanders may be assimilated by perpetrators and become new bullies (23, 24). Therefore, it is crucial to intervene in the psychological experiences of victims and bystanders and reduce the risk of them becoming new bullies. Traditional research has mostly focused on the negative experiences of victims (25, 26), but increasing evidence suggests that the psychological states of bystanders are also worthy of attention (27, 28). Specifically, victims, due to direct attacks, are prone to decreased self-efficacy and emotional exhaustion (29, 30), while bystanders, although not directly harmed, may fall into moral dilemmas and compassion fatigue, or remain in a state of anxiety due to fear of becoming the next target (31, 32). This role differentiation often directly influences the effectiveness of coping strategies, with victims tending toward emotion-oriented coping and bystanders relying more on instrumental strategies (33, 34). In this process, a single intervention strategy may not meet the needs of different role groups (35). Especially in the medical workplace, the tightness of team collaboration may cause bystanders’ emotional exhaustion to indirectly affect patient care quality, further amplifying the negative effects of bullying (36). However, in real life, emotional needs and instrumental strategies are difficult to satisfy simultaneously among colleagues, family, and leaders (37). Colleague groups, based on their professional roles, form interactions with core expectations of task collaboration and efficiency prioritization (38). In this context, instrumental strategies are more likely to be accepted, while excessive emotional demands may be viewed as efficiency losses (39). Given the professionalism and strong knowledge system of the medical field, family groups can provide high-quality emotional support, while instrumental strategies are difficult to implement (40). The core authority of leadership is based on instrumental value, and when employees seek emotional support from their superiors, it may trigger power distance sensitivity, while relying solely on instrumental strategies may exacerbate emotional exhaustion (41, 42). In recent years, the rise of generative AI has provided a new solution for bullied roles (43, 44). This study will focus on analyzing how generative AI can enhance the emotional needs and instrumental strategies of bullying roles in the medical workplace.

In the medical workplace, victims may avoid seeking interpersonal help due to concerns about professional retaliation (45), while generative AI provides a low-risk channel for confiding (46). Bystanders can obtain moral decision-making support through generative AI, reducing negative emotional consumption (47). Therefore, the rise of generative AI has brought significant changes to organizational communication patterns (48), and its information delivery methods may become an important factor in alleviating the poor psychological conditions of bullying roles. Traditional information delivery focuses on fact exchange and task collaboration (49), while emotional delivery, through semantic analysis and emotional computation, simulates human empathetic expressions, helping individuals vent emotions and rebuild psychological safety (50, 51). For example, AI counseling tools based on natural language processing can analyze language patterns to identify users’ emotional states and dynamically adjust response strategies (52). However, in the medical workplace, the action pathways of these two information delivery modes differ significantly. When AI adopts pure information delivery, it may reinforce the objective recording effect of bullying behavior, increasing victims’ cognitive reappraisal pressure (53). In contrast, emotional delivery modes can alleviate emotional exhaustion by generating soothing feedback (54). For instance, in the simulation experiment by Kliewer and Sosnowski (55), the use of emotionalized AI responses in bullying scenarios was shown to decrease cortisol levels in victims, while informational responses had no significant effects. However, existing AI intervention studies have primarily focused on general scenarios and have not designed differentiated information delivery strategies for different bullying roles.

Emotional exhaustion refers to a state of emotional resource overconsumption, extreme fatigue, and energy depletion experienced by individuals under work pressure (56, 57). It reflects the emotional depletion that individuals experience due to prolonged exposure to work pressure (58). Previous research has found that emotional exhaustion manifests differently across various bullying roles (15). Victims, due to continuous exposure to negative interpersonal interactions, exhibit a linear cumulative pattern of emotional resource consumption (59). In contrast, bystanders, although not directly subjected to attacks, experience compassion fatigue and moral dilemmas, leading to significant impacts on their healthy work states (31). Specifically, observing colleagues being bullied may activate bystanders’ psychological defense systems, leading to a chronic stress response and a slower rate of emotional exhaustion compared to victims, though the duration may be longer (60). Notably, individuals’ cognitive appraisal systems influence the emotional exhaustion of bullying roles (61, 62). Bystanders with high psychological resilience may reduce exhaustion risk by reconstructing the meaning of events, while victims with low self-efficacy may fall into learned helplessness (63). Therefore, this study introduces generative AI to explore how the interaction between generative AI’s information delivery methods and medical workplace bullying roles can alleviate emotional exhaustion.

Emotion event theory posits that emotions are complex psychological processes triggered by interactions between individuals and their environment, emphasizing that emotions are not merely passive reactions to external events but also reflections of subjective evaluations and coping strategies (64). Based on this theoretical framework, this study investigates how generative AI’s information delivery methods influence medical workplace bullying roles and subsequently alleviate emotional exhaustion. Furthermore, the professional information transmitted by generative AI can provide objective and reliable information to help individuals in the medical workplace more effectively assess and address bullying behaviors. This fact-based information delivery can reduce the uncertainty and subjective misinterpretation of information, thereby minimizing unnecessary emotional reactions caused by information asymmetry (65, 66). Additionally, the professional information from generative AI can offer suggestions and guidance to help victims address bullying behaviors in a more rational and constructive manner (43, 67). Moreover, the emotional information transmitted by generative AI can help individuals reconstruct the subjective meaning of emotional events, enabling them to manage emotional stress more positively (68). Emotional information delivery aims to trigger individuals’ emotional experiences and guide them to focus on the subjective meaning and internal impact of emotional events (69). For example, generative AI can express understanding and support to victims through natural language, helping them find emotional resonance and comfort (70). This emotionalized information delivery can help victims better release emotional pressure and reduce the emotional pain caused by bullying behaviors (71). Meanwhile, for bystanders, the emotional information delivered by generative AI can evoke empathy and moral responsibility, encouraging them to take active intervention actions. Through this emotionalized information delivery, generative AI can foster a more supportive and inclusive work environment in the medical workplace, thereby reducing emotional exhaustion at the team level. Through this emotional regulation mechanism, generative AI not only helps alleviate emotional exhaustion in the medical workplace but may also promote the long-term development of a healthy workplace culture and enhanced emotional resilience. Therefore, this study aims to reveal the mediating role of generative AI in the relationship between workplace bullying and emotional exhaustion through the lens of emotion event theory, providing new theoretical insights and practical guidance for psychological health management in medical organizations.

This study adopts Emotion Event Theory (64) as its core theoretical framework. EET posits that emotions are dynamic psychological processes initiated by discrete events, where subjective appraisals (rather than objective events alone) determine emotional outcomes. We prioritize Emotion Event Theory over alternative models (e.g., Job Demands–Resources Model) for two reasons.

First, The Emotion Event Theory emphasis on event-driven appraisals aligns with our experimental manipulation of workplace bullying as a concrete, role-specific event (victim vs. bystander scenarios). Whereas the Job Demands–Resources Model explains exhaustion as a function of chronic job demands and resources (58), the Emotion Event Theory better captures the immediate cognitive-emotional sequencing triggered by discrete bullying incidents—a critical mechanism given our scenario-based design.

Second, The Emotion Event Theory uniquely elucidates how external interventions (e.g., AI-generated information) reshape event appraisals. For victims, emotional AI inputs may mitigate threat appraisals by fostering affective coping (69); for bystanders, professional AI inputs may exacerbate moral conflict by heightening cognitive dissonance. The Job Demands–Resources Model lacks the granularity to model such role-contingent intervention pathways.

Based on the above analysis, this study proposes the following hypotheses:

*H1:* Medical workplace bullying roles have a significant impact on emotional exhaustion, with victims experiencing significantly higher levels than bystanders.

*H2:* The interaction between generative AI’s information delivery methods and medical workplace bullying roles has a significant impact on emotional exhaustion.

*H2a:* Under the influence of generative AI delivering professional information, bystanders experience increased emotional exhaustion.

*H2b:* Under the influence of generative AI delivering emotional information, victims’ emotional exhaustion is alleviated.

## 2 Methods

### 2.1 Participants

To test the four hypotheses, we designed a 2 (medical workplace bullying role: victim vs. bystander) × 2 (generative AI information delivery: professional information vs. emotional information) between-subjects experiment. We converted the paper questionnaires into electronic questionnaires and uploaded them to a professional data collection platform Credamo.^1^ We first contacted the human resource management departments of four hospitals in Nanchang city, Jiangxi Province, and Nanchong City, Sichuan Province. We explained the purpose of the study, the content of the study, the risks, the potential harms and the potential benefits to the director of the department. We obtained consent from the human resources department of the hospital, and the staff from the department assisted us in distributing the electronic and paper questionnaires together. We then randomly recruited 597 medical staff from four tertiary class A hospitals in Nanchang, Jiangxi Province, and Nanchong, Sichuan Province. We informed the participants about the purpose, procedure, and potential risks of the experiment, and all participants signed an informed consent form before starting the questionnaire. This study was approved by the Academic Ethics Committee of Jiangxi Normal University.

The inclusion criteria for this study were: (1) being a medical staff member; (2) working in a tertiary hospital; (3) having normal language communication skills and no obvious cognitive impairment; (4) not having participated in a similar study in the past month; (5) having used generative AI to query information and chat in the past month; and (6) completing the questionnaire within 10 min. The inclusion criterion (6) is due to the fact that our study uses the form of video and pictures for manipulation, where video and picture materials contain a lot of information and require a certain amount of time to understand and analyze. Therefore, in the process of questionnaire design, we set the playing time and presentation time of video and picture materials (2 min for picture materials and 2 min for video materials) to ensure that participants could effectively read and understand the responding materials. Regarding participants longer than 10 min, this is often seen with pausing and forgetting of patient questionnaires, which tends to reduce the accuracy of the data and should be eliminated.

The data collection period was from April to May 2025, and a total of 648 participants were recruited. After excluding 51 participants who did not meet the inclusion criteria (8 non-medical staff, 29 non-tertiary hospital staff, 12 participants took less than 5 min to respond, and 2 participants who had not used generative AI in the past month) (72), the effective sample size was 597, with an effective response rate of 92.12%.

In terms of demographic characteristics, there were 269 male participants and 328 female participants. The age distribution was: 18–25 years (12.9%), 26–40 years (52.1%), 41–60 years (29.6%), and 61 years and above (5.4%). The education level of the participants was: 82 (13.8%) with a junior college degree, 271 (45.4%) with a bachelor’s degree, 135 (22.6%) with a master’s degree, and 109 (18.3%) with a doctoral degree. Notably, the number of participating doctors was significantly lower than that of nurses, which may be due to the 1:2 doctor-to-nurse ratio in Chinese clinical hospitals, with a 1:4 ratio in key departments.

### 2.2 Experimental procedure

We randomly assigned all participants to either the victim or observer group. We asked all participants to imagine themselves in a medical workplace bullying scenario. We showed the victim group a video of workplace bullying in a medical setting, featuring only the bully and the victim. Meanwhile, we showed the observer group a video of workplace bullying from the observer’s perspective. We then asked both groups: “After watching the video, do you agree that you have had similar workplace experiences?” (1 = strongly disagree, 7 = strongly agree) (73). This question was used to assess the participants’ cognitive degree of workplace bullying. Video details are provided in Supplementary material.

In the victim group: The video shows a total of 2 participants, a man, the attending physician, and a woman, the nurse, in the operating room after the surgery. However, due to the work errors of the nurses, the operation time was prolonged, which increased the workload of the attending physicians. Consequently, the attending physician grabbed the nurse by the back of the head and verbally abused the nurse.

In the observer group: The video shows a total of 3 protagonists in the hospital corridor. The man in blue was the attending physician, and the two thin women were the nurses. Among them, a nurse’s work error caused an increase in the workload of the attending physician, which made the attending physician very angry. The attending physician beat and verbally scolded one of the nurses, while the other nurse witnessed the proceedings.

**Figure 1:**
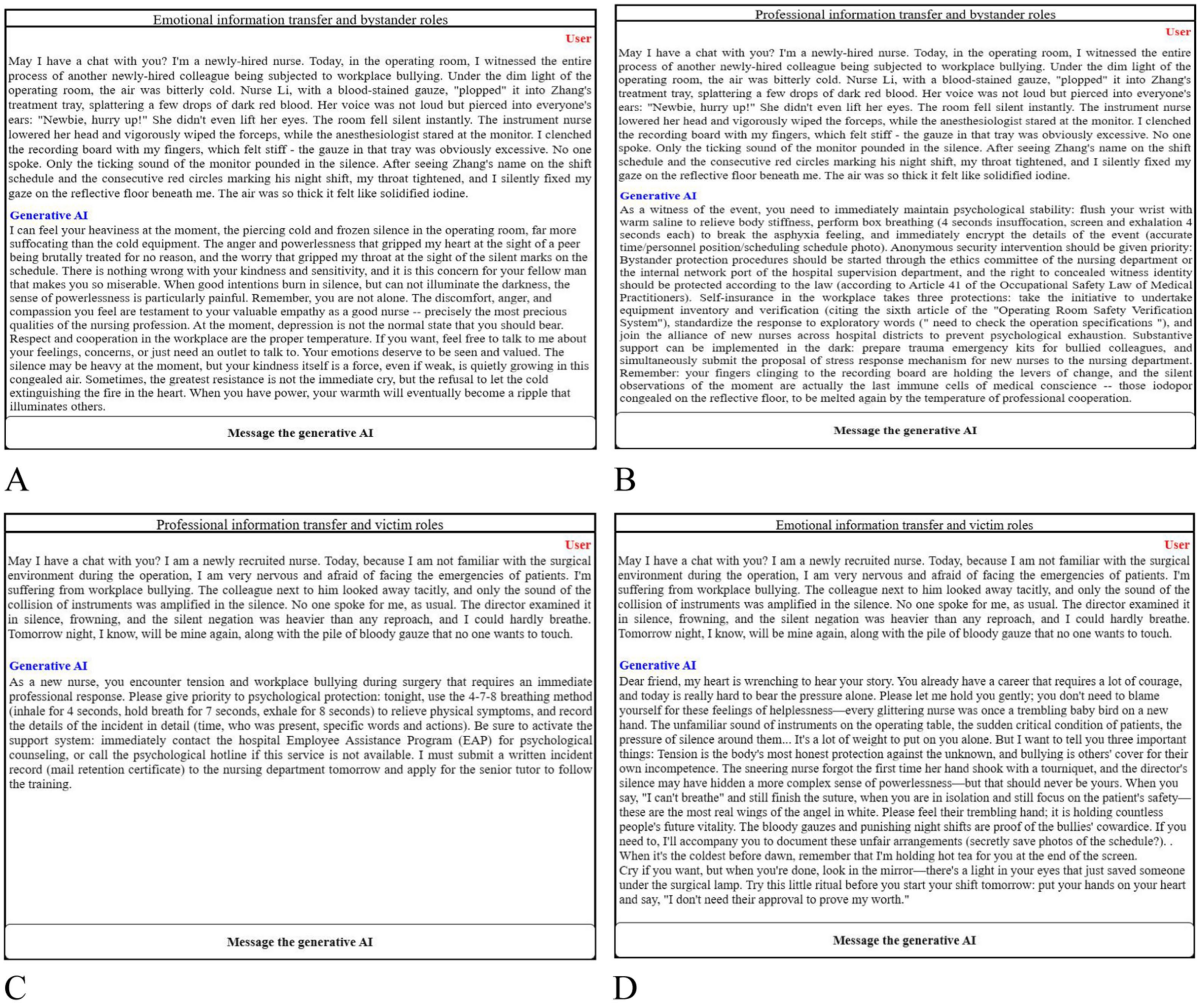
Stimulus map of the way information is transferred in generative AI. **(A)** Emotional information and bystander roles; **(B)** professional information and bystander role; **(C)** professional information and victim role; **(D)** Emotional information and victim role.

We then limited the generative AI’s response mode to either professional information or emotional information and asked the same question to the AI, obtaining different answers as the experimental stimulus materials. We guided both groups to use the generative AI and randomly assigned them to either the professional information or emotional information group. We showed the victim group’s two subgroups the professional information and emotional information stimulus materials, respectively. We asked the participants: “After reading the generative AI’s response, do you agree that your emotions have been comforted?” (1 = strongly disagree, 7 = strongly agree) (Figure 1).

Finally, participants completed the measurement questionnaires for work emotional exhaustion, adapted from Maslach and Jackson (74). The scale has been widely used in Chinese populations and has good cultural adaptability and reliability. In Xu et al. (75) research, the scale was translated into Chinese, and its cultural adaptability was verified in Chinese nurses. We used the scale’s eight measurement items to assess work emotional exhaustion, such as “Do you agree that your work makes you feel emotionally exhausted?” (1 = strongly disagree, 7 = strongly agree, Cronbach’s alpha = 0.834). We used AMOS29.0 software to verify the goodness of fit of variables and found that the model of work emotional exhaustion was well fitted (GFI = 0.986, AGFI = 0.972, RMSEA = 0.034, CFI = 0.998, TLI = 0.996). We also collected demographic information from the participants.

## 3 Results

Manipulation Check. Referring to Kline (76), we calculated the skewness and kurtosis of the role cognition and information emotionality. The results showed that the skewness and kurtosis values were less than 4 and 8, respectively, indicating that the data approximated a normal distribution (Skewness _role cognition_ = −0.66, Kurtosis _role cognition_ = −1.181; Skewness _information emotionality_ = −0.233, Kurtosis _information emotionality_ = −1.123). We then examined the manipulation effectiveness of medical workplace bullying role and generative AI information delivery, using role cognition and information emotionality as test variables, with an independent samples t-test. The results showed significant differences in role cognition [M _bystanders_ = 4.32, SD _bystanders_ = 2.404; M _victims_ = 5.26, SD _victims_ = 1.979; t (1,595) = 5.256, *p* < 0.001] and information emotionality [M _bystanders_ = 4.79, SD _bystanders_ = 2.248; M _victims_ = 4.49, SD _victims_ = 1.902; *t*(1,595) = 3.638, *p* < 0.001] between the victim and observer groups. Therefore, the manipulation of medical workplace bullying role and generative AI information delivery was successful.

Main Effect Analysis. We conducted a one-way ANOVA with medical workplace bullying role as the independent variable and work emotional exhaustion as the dependent variable. The results showed that the victim group’s work emotional exhaustion (M = 4.413, SD = 1.467) was significantly higher than that of the bystanders group [M = 4.729, SD = 0.997; *F*(1,595) = 9.512, *p* < 0.001]. This indicates that medical workplace bullying role has a significant impact on work emotional exhaustion, supporting Hypothesis 1.

**Figure 2:**
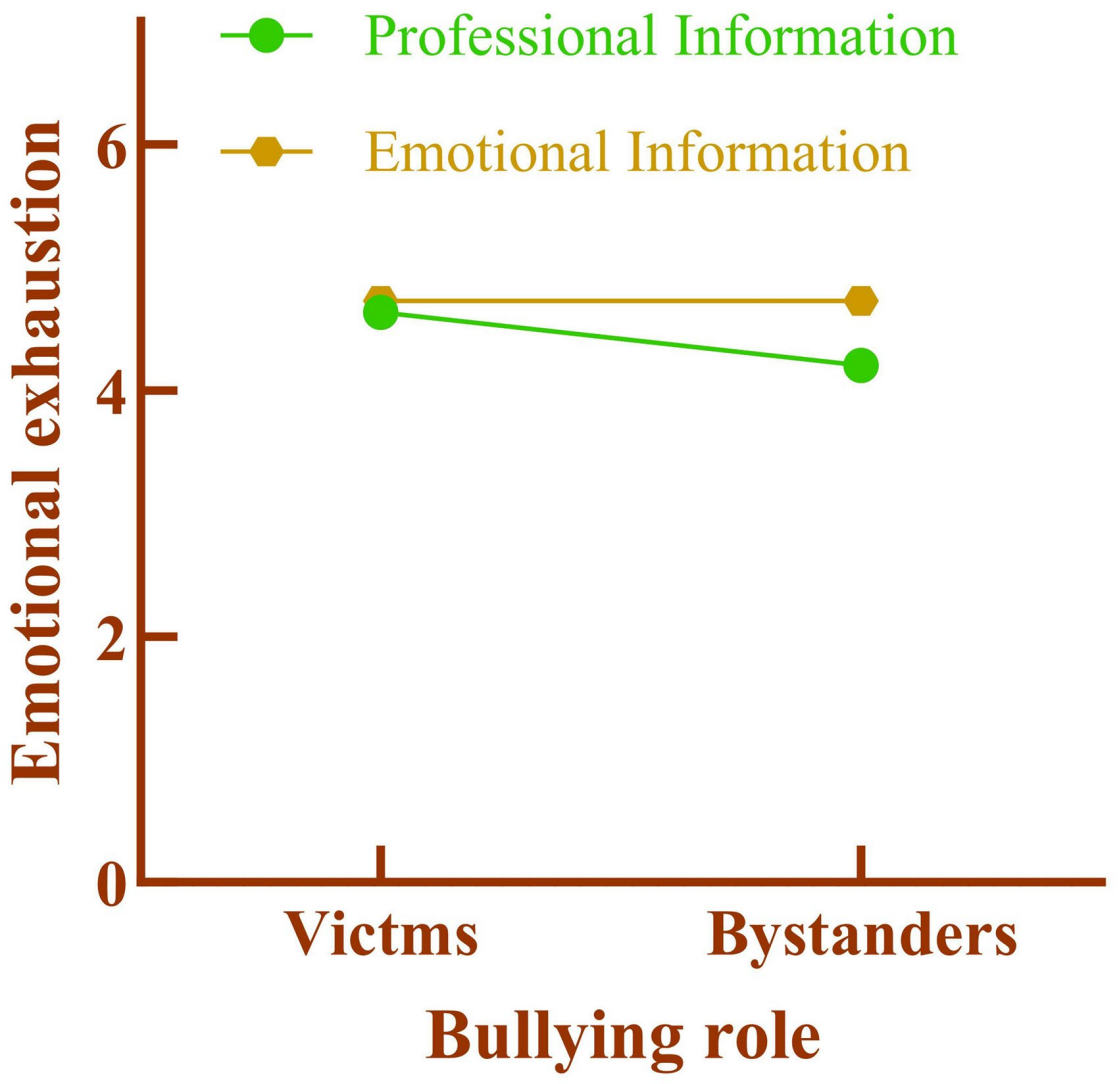
Interaction between generative AI messaging and bullying roles in the healthcare workplace.

Interaction Effect Test. We conducted a process model 1 analysis to examine the moderating effect of generative AI information delivery on the relationship between medical workplace bullying role and work emotional exhaustion. The results showed that medical workplace bullying role (*β* = 0.3156, 95% CI = [0.1155, 0.5157], *p* = 0.002) and generative AI information delivery (β = −0.2132, 95% CI = [−0.4133, −0.0131], *p* = 0.036) significantly predicted work emotional exhaustion. The interaction between generative AI information delivery and medical workplace bullying role also significantly predicted work emotional exhaustion (β = 0.4294, 95% CI = [0.0291, 0.8297], *p* = 0.0356). This indicates that generative AI has a significant moderating effect on the relationship between medical workplace bullying role and work emotional exhaustion, as shown in Figure 2, supporting Hypothesis 2.

Control Variable Analysis. Based on Schadenhofer et al. (77) study, which found that gender has a significant impact on medical staff’s emotional exhaustion, we conducted a one-way ANOVA to examine the effect of gender on work emotional exhaustion. The results showed that gender had no significant effect on work emotional exhaustion [*F*(1,595) = 0.123, *p* = 0.726]. Therefore, the effect of gender on the experimental results can be excluded.

In view of the research findings of Fan et al. (78), different identities of doctors and nurses may lead to differences in work stress, thus resulting in different effects of emotional exhaustion at work. Therefore, we used one-way ANOVA with professional role (doctor *Vs* nurse) as the independent variable and emotional exhaustion at work as the dependent variable. The results showed that professional role had no significant effect on work-related emotional exhaustion [*F*(1,595) = 1.421, *p* = 0.234]. Therefore, we excluded the influence of occupational role on the experimental results.

## 4 Discussion

### 4.1 Theoretical implications

This study explores the interaction between generative AI’s information delivery methods and the emotional exhaustion of bullying roles in the medical workplace, making significant theoretical contributions to organizational psychology and the application of artificial intelligence in workplace interventions. First, the findings expand the theoretical framework of workplace bullying by revealing the differential effects of various bullying roles on emotional exhaustion. While traditional research has primarily focused on the negative psychological consequences for victims (79, 80), this study, by comparing bystanders, found that their level of emotional exhaustion, although lower than that of victims, can still worsen when exposed to professional AI information. This highlights the complexity of workplace bullying’s impact, indicating that bystanders are not entirely immune to its negative effects. This calls for a more systematic examination of the dynamic psychological mechanisms across different bullying roles. Additionally, the results support the social cognitive theory, which posits that individuals’ perceptions and emotional reactions to their work environment are moderated by their role orientation (81, 82). This provides a new theoretical perspective for understanding the psychological adaptation processes in bullying contexts.

The study finds significant interactions between the information delivery methods of AI and bullying roles, suggesting that the effectiveness of AI interventions is not universal but highly dependent on the psychological state and role identity of the recipients. This aligns with the media synchronicity theory, which states that the effectiveness of information delivery depends on how well it matches the needs of the audience. Specifically, the alleviating effect of emotional information on victims may stem from its emotional support function, consistent with the core hypothesis of social support theory, which argues that emotional support reduces stress (83). Conversely, the negative impact of professional information on bystanders resonates with cognitive load theory, which suggests that highly professionalized information in high-pressure contexts may exacerbate psychological resource depletion (84). These findings provide theoretical support for the application of AI in organizational management, emphasizing the importance of customized information delivery strategies.

Emotional exhaustion, as a core dimension of job burnout, has traditionally been studied through the lens of the job demands-resources model (85). This study, however, introduces AI interventions to reveal the potential role of technological tools in regulating workplace psychological risks. The finding that emotional AI information alleviates victims’ emotional exhaustion aligns with affect regulation theory, which suggests that external emotional inputs can help individuals rebuild emotional balance (86, 87). Conversely, the negative impact of professional information on bystanders may stem from its failure to effectively meet their emotional needs and its potential to indirectly reinforce feelings of helplessness by emphasizing problem-solving. This extends the theory of emotional exhaustion interventions by proposing that technology-driven information delivery can complement traditional organizational interventions, but its effectiveness depends on precise alignment with the psychological characteristics of the target group.

### 4.2 Practical implications

The medical workplace is a high-pressure, high-intensity environment where healthcare professionals face demanding tasks and complex interpersonal relationships. This study demonstrates that workplace bullying in the medical field significantly impacts employees’ mental health, particularly in terms of victims’ emotional exhaustion. Therefore, hospitals should establish and improve anti-bullying policies by conducting regular training, anonymous surveys, and psychological counseling to enhance employees’ awareness of workplace bullying and reduce its occurrence. Additionally, hospitals should prioritize the integration of generative AI technologies, particularly AI systems with emotional support capabilities, to provide employees with psychological support and emotional relief resources. In terms of professional information delivery, AI systems can help employees complete tasks more efficiently, thereby reducing workload pressure. In terms of emotional support, AI systems can offer victims timely psychological comfort and resource guidance. Hospital management should also foster an open and inclusive organizational culture by encouraging supportive communication among employees, reducing feelings of isolation and helplessness, and thereby lowering the risk of emotional exhaustion.

As an emerging technology, the application of generative AI in the medical workplace requires a balance between emotional support and professional information delivery. This study finds significant differences in how AI’s information delivery methods alleviate emotional exhaustion among medical professionals. This suggests that AI designers should optimize its information delivery functions to flexibly adjust the nature of the information based on the needs of different roles. For instance, when interacting with victims, AI systems should provide more emotional support and psychological comfort. When engaging with bystanders, AI systems should offer professional support while avoiding purely factual information transmission to prevent worsening emotional burdens. Furthermore, generative AI should possess interaction capabilities to sense users’ emotional states and automatically adjust its information delivery methods to better meet diverse user needs.

This study shows that victims of workplace bullying in the medical field experience significantly higher levels of emotional exhaustion than bystanders. Victims often need to take active measures to address bullying behaviors and alleviate emotional exhaustion. Victims can proactively utilize generative AI technology to obtain psychological comfort and resource suggestions through its emotional support functions. For example, AI systems can provide victims with access to psychological counseling resources, legal support channels, and assistance pathways within and outside their organizations. Additionally, victims should learn to actively seek support rather than endure the situation in silence. Support systems within hospitals and positive interactions among colleagues are crucial pathways to alleviating emotional exhaustion.

Bystanders play a critical role in workplace bullying in the medical field, but this study finds that the professional information delivery methods of generative AI may exacerbate bystanders’ emotional exhaustion. Therefore, bystanders should actively learn to become positive influencers by offering support and resources to victims, thereby reducing their own emotional burdens. Based on the study’s findings, bystanders can utilize the emotional support features of generative AI to alleviate the emotional distress caused by witnessing bullying behaviors. At the same time, bystanders need to learn to regulate their emotional responses rationally, avoiding feelings of helplessness that may arise from purely factual information transmission.

### 4.3 Limitations and future research directions

Despite the aforementioned findings, this study has several limitations. First, this study acknowledges critical limitations in geographical and cultural generalizability. Our sample was exclusively drawn from hospitals in Nanchang and Nanchong, which represent urban centers in Central and Western China. While these regions exhibit typical characteristics of China’s high-pressure medical systems, the findings may not fully generalize to: (1) Rural healthcare settings with distinct resource limitations; (2) Hospitals in Eastern Chinese megacities where organizational cultures may differ; (3) Non-Chinese contexts where cultural norms modulate bullying dynamics and help-seeking behaviors. For instance, Confucian values prevalent in China may amplify bystanders’ moral conflict when witnessing authority figures engage in bullying, potentially intensifying the negative effect of professional AI information observed in our study. Conversely, cultures with lower power distance might show attenuated effects. Future research should prioritize multi-regional sampling within China and comparative designs across cultures to disentangle cultural from systemic factors.

Second, this study primarily discusses the transmission of professional and emotional information but does not delve into the issue of individualized adaptation of generative AI across different workplace roles. For example, can the emotional support functions of generative AI dynamically adjust based on individual differences? Future research can further optimize generative AI algorithms to enable more personalized information delivery based on users’ actual needs and emotional states, thereby more effectively alleviating emotional exhaustion. Additionally, whether generative AI’s information delivery may influence other roles in the medical workplace is another direction worth exploring.

Third, this study employs a cross-sectional research design, which only captures the static relationship between variables at a single time point and cannot reveal the long-term dynamic mechanisms by which generative AI’s information delivery methods interact with medical workplace bullying roles. For example, can the emotional support functions of generative AI sustainably alleviate victims’ emotional exhaustion over long-term use? Can the professional information transmission of generative AI produce cumulative effects on bystanders’ emotional states? To address these questions, future research can adopt a longitudinal design to examine the long-term impact of different information delivery methods of generative AI on emotional exhaustion among medical professionals. Additionally, this study measures emotional exhaustion using self-report questionnaires, which may introduce subjective biases. Future research could incorporate physiological indicators (e.g., skin conductance, heart rate variability) or objective behavioral data (e.g., work efficiency, error rates) to validate changes in emotional exhaustion.

Fourth, while our scenario-based experiment effectively captured immediate emotional responses to AI interventions, the cross-sectional design inherently limits insights into long-term effects. According to the Conservation of Resources Theory (88), emotional recovery from bullying requires sustained resource replenishment. For victims, repeated exposure to AI-driven emotional support may gradually rebuild psychological capital through mechanisms such as habitual emotion regulation. Conversely, bystanders’ cumulative exposure to professional AI information could exacerbate cognitive load over time, potentially leading to chronic emotional detachment. Future longitudinal studies should track how frequency of AI usage and intervention durability jointly shape emotional trajectories across bullying roles.

Fifth, our measurement of emotional exhaustion relied on self-reported data collected immediately after participants viewed emotionally charged bullying videos. While this design aligns with Emotion Event Theory’s focus on immediate cognitive-emotional sequencing (64), the responses may reflect transient affective states rather than stable emotional exhaustion. Future studies should triangulate self-reports with physiological measures (e.g., cortisol levels, heart rate variability) to capture objective stress responses. Longitudinal designs tracking emotional exhaustion over time could further disentangle short-term reactions from chronic depletion.

Finally, while this study reveals the potential role of generative AI in mitigating emotional exhaustion caused by workplace bullying in the medical field, its practical application still faces challenges. For instance, could generative AI’s information delivery raise concerns about privacy and data security among employees? Can the emotional support functions of generative AI gain acceptance and trust from employees? Future research can conduct field-based empirical studies to examine the practical effects of generative AI in real-world medical workplaces and explore its applicability across different cultures and organizational environments. Furthermore, this study did not examine other potential influencing factors in the medical workplace, such as organizational culture, leadership styles, and team collaboration. Future research can incorporate these variables into the model to develop a more comprehensive theoretical framework.

## 5 Conclusion

This study conducted a scenario-based experiment with 597 participants to verify the impact of generative AI’s information delivery methods and their interaction with medical workplace bullying roles on emotional exhaustion. Specifically, the findings show that under the influence of professional information delivery by generative AI, bystanders experience increased emotional exhaustion. Conversely, under emotional information delivery by generative AI, victims’ emotional exhaustion is alleviated. This research not only expands the application of emotion event theory but also advances the progress of anti-bullying initiatives in organizations, providing specific suggestions and practical references.

## Data Availability

The original contributions presented in the study are included in the article/Supplementary material, further inquiries can be directed to the corresponding authors.

## Footnotes

^1^ https://www.credamo.com/

## Supplementary material

Supplementary Video 1 Victim’s perspective: Two participants are the perpetrator and the victim.

Supplementary Video 2 Bystander perspective: The three participants are bystander, victim, perpetrator.
