# Correction: The impact of generative AI'S information delivery methods on emotional exhaustion among bullying roles in the medical workplace

**DOI:** 10.3389/fpubh.2025.1724549

**Published:** 2025-10-24

**Authors:** Lihong Deng, Dajun Yang, Gongzhuoran Liang, Chang Hu, Pengcheng Zhang

**Affiliations:** ^1^College of Management, North Sichuan Medical College, Nanchong, China; ^2^Key Laboratory of Digital Intelligent Disease Surveillance and Health Governance, North Sichuan Medical College, Nanchong, China; ^3^Sichuan Provincial Primary Health Service Development Research Center, North Sichuan Medical College, Nanchong, China; ^4^College of Physical Education, Jiangxi Normal University, Nanchang, China

**Keywords:** generative AI, information delivery methods, workplace bullying, bullying roles, emotional exhaustion, bystander, victim

Affiliations 1 and 2 were erroneously given as:

^1^ College of Management, Guanbei Medical University, Nanchong, China

^2^ Key Laboratory of Digital-Intelligent Disease Surveillance and Health Governance, Guanbei Medical University, Nanchong, China

The corrected affiliations 1 and 2 are:

^1^College of Management, North Sichuan Medical College, Nanchong, China

^2^Key Laboratory of Digital Intelligent Disease Surveillance and Health Governance, North Sichuan Medical College, Nanchong, China

The original version of this article has been updated.

